# Importance of Maternal Diabetes on the Chronological Deregulation of the Intrauterine Development: An Experimental Study in Rat

**DOI:** 10.1155/2015/354265

**Published:** 2015-02-09

**Authors:** Marcela Salazar García, Elba Reyes Maldonado, María Cristina Revilla Monsalve, Laura Villavicencio Guzmán, Alfonso Reyes López, Concepción Sánchez-Gómez

**Affiliations:** ^1^Laboratorio de Investigación en Biología del Desarrollo y Teratogénesis Experimental, Hospital Infantil de México Federico Gómez, Dr. Márquez 162, 06720 Colonia Doctores, DF, Mexico; ^2^Escuela Nacional de Ciencias Biológicas, Instituto Politécnico Nacional, Prolongación de Carpio y Plan de Ayala, 11340 Colonia Santo Tomas, DF, Mexico; ^3^Unidad de Investigación Médica en Enfermedades Metabólicas, Centro Médico Nacional Siglo XXI, Instituto Mexicano del Seguro Social, Avenida Cuauhtémoc 330, 06725 Colonia Doctores, DF, Mexico; ^4^Dirección de Investigación, Hospital Infantil de México Federico Gómez, Dr. Márquez 162, 06720 Colonia Doctores, DF, Mexico

## Abstract

We investigated whether maternal diabetes induced in rats using streptozotocin (STZ) on Day 5 of pregnancy affects the intrauterine developmental timeline. A total of 30 pregnant Sprague-Dawley diabetic rats (DRs) and 20 control rats (CRs) were used to obtain 21-day fetuses (F21) and newborn (NB) pups. Gestational age, weight, and body size were recorded as were the maxillofacial morphometry and morphohistological characteristics of the limbs. In DRs, pregnancy continued for ∼1.7 days, and delivery occurred 23 days postcoitus (DPC). In this group, the number of pups was lower, and 13% had maxillofacial defects. F21 in the DR group had lower weights and were smaller; moreover, the morphological characteristics of the maxillofacial structures, derived from the neural crest, were discordant with their chronological gestational age, resembling 18- to 19-day-old fetuses. These deficiencies were counterbalanced in NB pups. We conclude that hyperglycemia, which results from maternal diabetes and precedes embryo implantation, deregulates the intrauterine developmental timeline, restricts embryo-fetal growth, and primarily delays the remodeling and maturation of the structures derived from neural crest cells.

## 1. Introduction

Clinical and experimental evidence suggests that maternal diabetes that is poorly controlled from the beginning of pregnancy often causes embryonic or fetal death [1–4] and congenital diseases of various organs, which are common causes of prenatal death [4–16]. In less extreme cases, maternal hyperglycemia disturbs prenatal growth and development, leading to the development of macrosomia or microsomia [17–20]. Human studies investigating the mechanisms responsible for the deleterious effects of maternal diabetes on the offspring remain partial and limited. These limitations are partially because it is not possible to control the variables that change the intrauterine environment in humans or analyze their effects on various aspects of embryo-fetal development. The rat is the biological model of choice to address the topic for the following reasons: (1) diabetes can be induced in a regulated manner [21]; (2) their embryonic, anatomic, and physiological characteristics are similar to those of humans [22]; and (3) their life cycle and gestational period are relatively short. Based on these reasons, the current study investigated whether maternal diabetes induced in rats using streptozotocin (STZ) before embryo implantation, therefore presiding morphogenesis, in addition to causing congenital malformations, affects the intrauterine developmental timeline. For this purpose, we analyzed the precise length of gestation; the anthropometric and maxillofacial characteristics of perinatal pups from diabetic rats (DRs) and control rats (CRs) were analyzed.

## 2. Materials and Methods

Pregnant Sprague-Dawley rats were randomly separated into two large groups. Diabetes was induced in the first group during the preimplantation embryo stage (5 DPC) to create DRs. The second group remained untreated and was used as CRs. A total of 30 DRs and 20 CRs were used to obtain one hundred 21-day normal fetuses (F21) and 100 newborn (NB) normal pups with harmonic axial symmetry and a premaxilla that was more prominent than the mandible. In addition to gestational age (day postcoitus) weight and body size were determined, as was the morphometry of the maxillofacial structures and histological characteristics of the limbs for all specimens. The animal use protocols and study procedures were strictly based on the Mexican Official Standard (NOM-062-ZOO-1999) [23]. In addition, the research, ethics, and biosafety committees of the Children's Hospital Federico Gomez of Mexico City approved this project.

### 2.1. Acclimatization

Sexually mature Sprague-Dawley rats weighing between 250 and 300 g were obtained from the Animal Facilities of the National Medical Center Siglo XXI, Mexican Institute of Social Security (IMSS, for its acronym in Spanish). Females and males were acclimatized for 2 weeks prior to mating in separate cages and under controlled lighting (12-h/12-h light/dark cycles), humidity (50% ± 10%), and temperature (22 ± 2°C) conditions.

### 2.2. Cycle Determination and Mating

After the acclimatization period, vaginal smears stained with 0.5% toluidine blue were used to determine the day on which females were receptive to the male. Blood and urine glucose levels were determined in females during the estrus stage. A group of three rats with these characteristics were placed in a cage with a male where they remained for approximately 12 hours (7 pm to 7 am). Pregnancy was confirmed by the presence of a vaginal mucus plug, sperm in the vaginal smear, or both; this evidence marked this date Day 0 of pregnancy. Pregnant rats were placed in individual cages under controlled conditions similar to those of the acclimatization period.

### 2.3. Induction of Diabetes

The diabetes-inducing method was based on a protocol described in previous studies [9, 24]. Briefly, on the fifth day of pregnancy (preimplantation period) STZ was injected intraperitoneally (Sigma, St. Louis, MO, USA) in a single 50-mg/kg dose. The experimental group only included rats whose blood glucose levels were ≥200 mg/dL 48 h after the administration of STZ. From the beginning of pregnancy until sacrifice, body weight, blood glucose, and glycosuria were measured in both groups, and special care was taken to record the length of gestation.

### 2.4. Weight and Somatometry

F21 that were obtained via Cesarean section and NB pups obtained by spontaneous delivery were sacrificed with ether vapors, fixed in 4% formalin, and weighed on an electronic balance scale (Chyo JL-180). Using a digital Vernier caliper (Starrett), the crown-rump length, nasooccipital length, tail length, and waist circumference at the umbilicus were measured. In both age groups (F21 and NB) the number of individuals per litter was registered. Both fetuses and newborns were morphologically evaluated. Those showing any external malformation were excluded from the analysis of weight and somatometry.

### 2.5. Morphological Analysis

To determine whether any difference existed in the external morphological characteristics of the maxillofacial structures and limbs, both the malformed and normal fetuses and NBs of DRs and CRs were analyzed using a Lumar V12 stereomicroscope (Zeiss), with special emphasis on the development of the eyelids, ears, and interdigital webbing. Images were captured using an Axiocam MRc microscope digital camera (Zeiss). In addition, 10 forelimbs and 10 hind legs from each group, previously fixed in 4% formalin, were processed to determine their histological characteristics. These specimens were made transparent with cedar oil and embedded in VIP (Sakura Finetek, Torrance, CA, USA) paraffin. Serial 5-*μ*m thick histological sections in the sagittal plane were prepared along the palmar and plantar surfaces and stained with Hematoxylin and Eosin. An equal number of limbs were fixed in 2.5% glutaraldehyde and processed for analysis with a scanning electron microscope. After dehydration in a graded series of alcohols, these samples were prepared via critical point drying in a Samdri 789^a^ (Tousimis Rockville MD) apparatus and shadowed with a 350-nm thick gold layer in a Denton Vacuum Desk 1A apparatus (Cherry Hill Industrial Centre, NJ, USA). The samples were observed at 15 kV in a JEOL JSM 5300 scanning electron microscope (JEOL, Tokyo, Japan).

### 2.6. Statistical Analyses

Changes in weight, glycemia, and glycosuria were evaluated throughout gestation using two-factor linear models with repeated measures on one factor. The Bonferroni adjustment was used for pairwise comparisons. In addition, the effects of diabetes on the variables recorded in the fetuses and NB pups were analyzed with a two-way ANOVA using *α* = 0.05. The data were analyzed with SPSS version 15.0.

## 3. Results

### 3.1. Gestation Period

Gestation lasted 21 days in CRs. The gestation of DRs continued for 1.7 days on average; therefore, spontaneous delivery occurred 23 days postcoitus (Table 1).

### 3.2. Metabolic Control and Maternal Body Weight

In concordance with literature [24], high glucose levels in the blood and urine of the diabetes-induced (via STZ) pregnant rats predominated throughout the study. Two days after treatment with the drug, blood glucose levels in the DRs were ≥200 mg/dL (Table 2), and glycosuria reached 1,000 mg/dL. In both cases, the values increased gradually during pregnancy and often doubled. In contrast, the blood glucose levels of CRs were just above 105 mg/dL (Table 2), and glycosuria was not detected.

CRs showed exponential increases in body weight during pregnancy. However, pregnant DRs showed less overall weight gain, with a reduction in weight gain 15 DPC (Table 3).

### 3.3. Chronological Age of the Gestation Products

Both the F21 and NBs of the CRs were obtained 21 DPC. Fetuses were acquired by Cesarean section between 8 am and 9 am, and NBs were obtained via spontaneous delivery, usually after 12 pm. In contrast, the F21 of DRs were obtained via Cesarean sections on the morning 21 DPC, whereas their NBs were spontaneously delivered 23 DPC, usually during the evening.

### 3.4. Litter Characteristics

**Figure 1:**
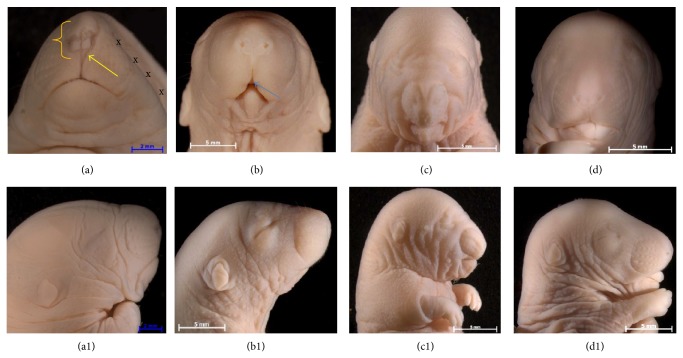
The common craniofacial malformations associated with maternal hyperglycemia. ((a), (a1)) Frontal and sagittal views show hemifacial microsomia with bilateral asymmetry of the left side, malformed facial muscles and bones (xxx), lateral facial cleft (yellow arrow), and bifid nose (bracket). ((b), (b1)) Notice cleft lip-palate (blue arrow) and agnatia. ((c), (c1)) Observe maxillary agenesis. ((d), (d1)) It shows macroglossia. ((a1), (c1), and (d1)) Notice the hypodeveloped eyelids and the nonopen nostrils.

DRs had fewer pups as well as more resorptions and intrauterine deaths compared with CRs (Table 1). Additionally, 100% of CR fetuses showed normal morphology, whereas 2% of the DRs products depicted limb defects and 13% showed craniofacial malformations sharing characteristics with the oculoauriculovertebral syndrome as observed in children of diabetic mothers. It was common to observe facial immaturity manifested by macroglossia (Figures 1(d) and 1(d1)), hypodeveloped eyelids, and nonperforated nostrils (Figures 1(a1), 1(c1), and 1(d1)). The relationship of the size and position of the mandible body with respect to the maxilla allowed diagnosing micrognathia and agnathia (Figure 1(b1)). Some DRs products showed a very pronounced lateral facial cleft, bifida nose, and cleft lip and palate (Figure 1(a)).

### 3.5. The Effects of Hyperglycemia on Normal F21 and NBs

#### 3.5.1. Weight and Somatometry

Although the fetuses of both DRs and CRs were obtained in the morning 21 DPC, the weight (6.27 g versus 3.61 g), crown-rump length (4.05 cm versus 3.30 cm), nasooccipital length (1.67 cm versus 1.34 cm), tail length (1.53 cm versus 1.34 cm), and waist circumference (1.45 cm versus 1.13 cm) were significantly lower among the morphologically normal fetuses of the DRs compared with those of the CRs (Table 4). However, these variables did not significantly differ when comparing the normal NB pups of CRs obtained in the evening 21 DPC and the normal NB pups of DRs obtained 23 DPC (Table 4).

**Figure 2:**
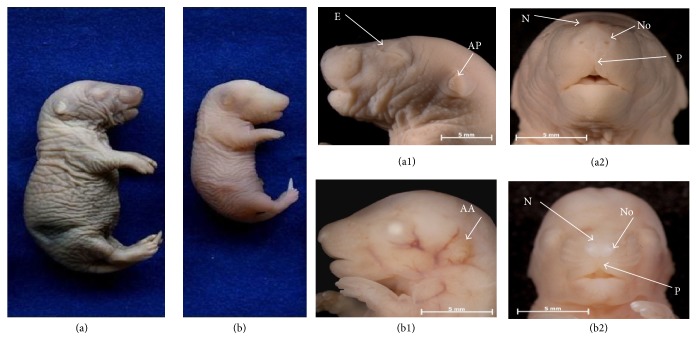
The body and facial morphology of control rat ((a), (a1), and (a2)) and streptozotocin-induced diabetic rat ((b), (b1), and (b2)) fetuses after 21 days of gestation. Note the difference in size of the fetus from the diabetic mother compared with the control and the marked immaturity of the maxillofacial structures. AA = auricular appendage, E = eyelids, AP = auricular pavilion, No = nostrils, N = nose, and P = philtrum.

The F21 of the DR group that did not show external malformations, despite having harmonic axial symmetry, showed external morphological characteristics that did not correspond to their chronological gestational age, the most obvious being their smaller size (Table 4, Figure 2(b)) and the less developed maxillofacial structures, eyes and ears (compare Figures 2(a) and 2(b)).

**Figure 3:**
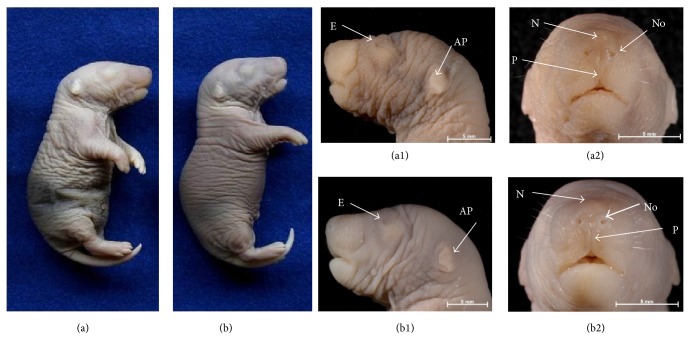
The body and facial morphologies of newborn pups obtained from control rats ((a), (a1), and (a2)) and streptozotocin-induced diabetic rats ((b), (b1), and (b2)). Note in the pup from the diabetic mother (b1) the medial surface of the helix with the straight edge in the auricular appendage. E = eyelids, AP = auricular pavilion, No = nostrils, N = nose, and P = philtrum.

Although the nasomedial processes were already fused, we found that nose reshaping was still emerging. The nostrils were not open, the philtrum was short, and the ala and the tip of the nose could not be distinguished. The lenses were completely opaque, and eyelid development was still emerging (Figures 2(b1) and 2(b2)). The auricular appendages were shaped as an amorphous fold with relatively low implantation (Figure 2(b1)). In contrast, the F21 and NBs of CRs obtained 21 DPC and the NBs of DRs acquired 23 DPC had similar external morphological features, all of them showing harmonic axial symmetry and a premaxilla that was more prominent than the mandible (Figures 2(a), 3(a), and 3(b)). In these cases, the nostrils were open, and the philtrum was well developed; in addition, the eyelids were already fused. The ears of the F21 and NBs from CRs were normally implanted with a curved medial surface of the helix; however, the edge was straight in the NBs of DRs (compare Figures 3(a1) and 3(b1)).

#### 3.5.2. Limb Morphology

**Figure 4:**
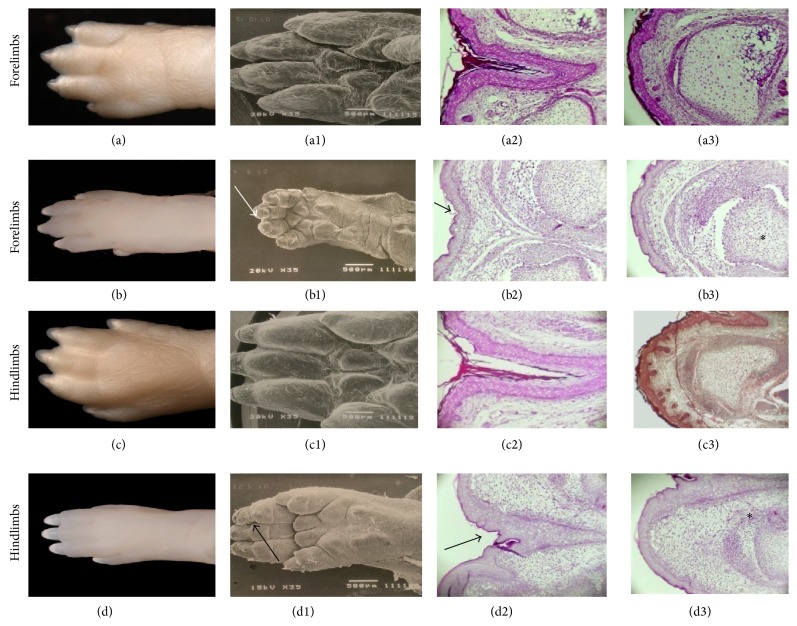
The morphohistology of the palmar and plantar surfaces of the limbs of F21 obtained from control rats ((a), (c)) and diabetic rats ((b), (d)). Note the minimal digit separation in F21 of DR (arrows in (b1), (b2), (d1), and (d2)) and the less advanced ossification process (^*^in (b3), (d3)).

**Figure 5:**
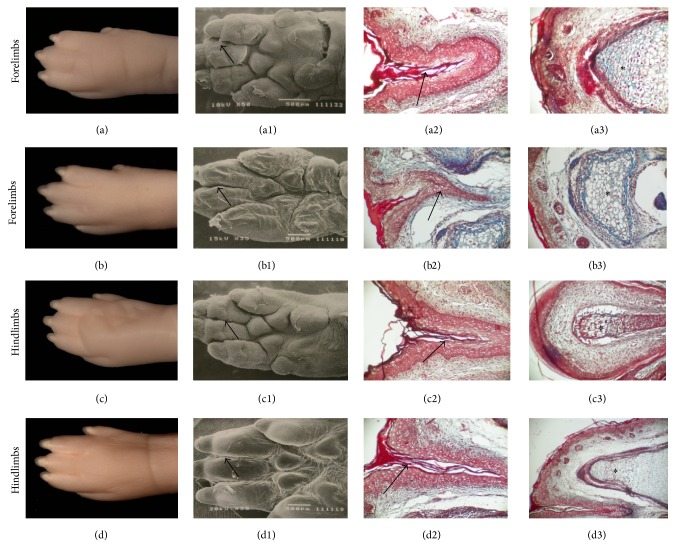
The morphohistology of the limbs of newborn pups obtained from control rats ((a), (c)) and diabetic rats ((b), (d)). Note that the interdigitating (arrows) and ossification (^*^) processes were similar in both study groups.

The normal offspring of the CRs (both F21 and NBs) did not show limb defects, whereas 2% of the apparently normal DR offspring presented oligodactyly with the absence of one or more digits. As expected, the forelimbs of the offspring of CRs were slightly more developed than their hind legs. In contrast, the F21 of DRs showed hind legs slightly more developed than the forelimbs (Figures 4(b1) and 4(d1)). Limb morphology was similar among the F21 and NBs of CRs (obtained during Day 21 of pregnancy) and the NBs of DRs (obtained via spontaneous delivery 23 DPC); in these cases digit separation was practically complete and the endochondral ossification of digits was significantly advanced (Figures 4(a), 4(c), and 5). In contrast, DR fetuses showed shorter limb length height and less maturation of the phalanges, fingernails, and pads (compare Figures 4(b1) and 4(d1) with 4(a1) and 4(c1), resp.). The histological analysis showed poor ossification of the phalanges and minimal digit separation because of the presence of the interdigit webbing (Figure 4).

## 4. Discussion

The gestational (GD) and pregestational (PGD) maternal diabetes have differential effects in the intrauterine development. The first is manifested toward the third trimester of pregnancy with levels of serum glucose ≥126 mg/dL. While this maternal disease does not cause severe birth defects, in both children and animal models it has been correlated directly with perinatal problems such as macrosomia, low birth weight, respiratory distress, and endocrinological defects. In contrast, the PGD is manifested before conception by high levels of serum glucose ≥200 mg/dL. It may affect the organogenesis from embryos and therefore disturb various aspects of the intrauterine development. At present, pregestational diabetes mellitus has considerably increased worldwide. This expansion directly correlates with high difficulty to conceiving, greater number of spontaneous abortions, fetoplacental disorders, perinatal mortality, and major congenital malformations. Despite these facts, no consensus exists regarding the morphogenetic mechanisms and molecular networks that underlie the effect of an early hyperglycemic intrauterine environment and its short- and long-term influences on offspring. This lack of data precludes significant progress regarding the prevention, early diagnosis, and proper treatment of the adverse effects of maternal diabetes in offspring. Due to ethical issues involving human studies that sometimes have invasive aspects and the multiplicity of uncontrolled variables that can alter the uterine environment during clinical studies, it is necessary to use animal models to better understand diabetic pathophysiology. Based on this matter, the current study experimentally examined whether induced diabetes in pregnant rats (prior to organogenesis) deregulates the embryonic development timeline. Furthermore, it sought to determine whether diabetes affects the morphological characteristics of term fetuses and NB pups. The most intriguing results were the long period of gestation in DRs and the marked variations in fetal maturation between the F21 of the control and diabetic groups.

### 4.1. Pregnant Rats and Liters Outcome

In accordance with the procedures described in the literature [8, 24], diabetes was induced in pregnant rats via the administration of STZ 5 DPC. Maternal disease was confirmed 48 h after drug administration using high serum glucose levels, which were often above 300 mg/dL (Table 2), and the presence of glycosuria. Like Eriksson et al. [25] and Kiss et al. [26] who used a similar animal model, we found that pregnant rats treated with STZ showed the typical features of severe diabetic status, with fatigue and reduced body weight gain (despite increased food intake), unlike pregnant controls. In line with Damasceno et al. [27], we assumed that these effects were the result of the metabolic imbalance (hyperglycemia) and hormonal imbalance (hypoinsulinemia) caused by diabetes, coupled with the hormonal changes of pregnancy. Although the higher resorption and intrauterine deaths rates might have contributed to reducing the weight gain in DRs, the case that 13% of their products showed severe craniofacial malformations rather supports the proposal of Yang et al. [28], who point out that lowered weight gain during human pregnancy may be a consequence of carrying a neural tube affected fetus or a marker for underlying factors common to the etiology of these birth defects. The fact that offspring displayed craniofacial malformations in DRs and no malformations in CRs confirms the normality of the rat strain while resistance to craniofacial defects caused by diabetic pregnancy is a genetic trait of the embryos.

### 4.2. Length of Gestation

It has been suggested that the metabolic and physiological changes caused by maternal hyperglycemia during pregnancy alter the length of gestation by modifying the energy balance of the embryo [29–31]. However, studies on the effects of maternal diabetes on labor induction in addition to being rarely addressed have shown controversial results. In humans, it is impossible to determine with certainty whether maternal diabetes modifies the length of gestation by the difficulty of accurately establishing the duration of this process. Although this parameter can be determined in animal models, most studies report their results using products obtained by Cesarean section 19 to 21 DPC [31]. Even studies that analyze the effects of maternal diabetes in NBs obtained via spontaneous work [30] do not usually specify the length of gestation. We induced severe hyperglycemia at 5 DPC just before the implantation of the embryo and beginning of the organogenesis and accurately registered the duration on pregnancy and in accordance with Eriksson et al. [32] and Mulay and Solomon [33] we found that gestation in DRs continued for approximately 2 days more and the number of pups was almost always lower than that of controls. In contrast, Amri et al. [29], who induced diabetes in early pregnancy by injecting 35 mg/kg of STZ and intravenously administering 30% hypertonic glucose 8 DPC to maintain blood glucose levels of ~218 mg/dL, reported that delivery was anticipated in 12 h. We suppose that variation in results depends largely on the experimental design used to induce diabetes, the period in which hyperglycemia is manifested (pregestation, postconception, and middle or late gestation), and the blood glucose level reached.

### 4.3. Developmental Delay

As expected, the F21 and NBs of the control group had the same chronological age and external morphological characteristics typical of their perinatal period with similar weights and heights (Table 4; Figures 2(a) and 3(a)). In contrast, the F21 and NBs of the DR group showed differences in morphology (Figures 2(b) and 3(b)) and somatometry (Table 4) consistent with the variations in chronological age (21 versus 23 DPC). Although gestation lasted approximately 2 days longer for DRs, the external morphologies, weights, and sizes of their NB pups were virtually identical to the F21 and NBs of the CRs (Table 4; Figures 2(a) and 3). In contrast, neither the anthropometric nor the maxillofacial characteristics of the F21 were in accordance with their chronological age; despite having lower weights and heights, they were morphologically similar to fetuses 17-18 DPC, as reported by Christie [34] and Marcela et al. [35]. Like Streck et al. [36], we found that DR F21 showed developmental limbs delays in both the long bones and the digits (Figures 4(b) and 4(d)). Likewise, the processes of remodeling and maturation concerning the structures derived from neural crest cells as the maxilla, mandible, nose, eyelids, and auricular appendages were not yet complete (Figure 2(b)). These results suggest that prolonged pregnancy time in DRs was essential for F21 to continue developing the immature structures.

When analyzing the effect of maternal hyperglycemia on fetuses 15–20 DPC, several studies have indicated that maternal diabetes causes delays in the fetal growth of various organs, including the pancreas and the placenta [30, 32, 37, 38], based on their weights and heights but ignoring the degree of fetal and organic maturation. However, our somatometric and morphological results do not support this claim. We consider that DR F21 are not only small but also immature because they have only achieved an intrauterine development equivalent to a 17- to 18-day-old fetus. All these evidences lead us to conclude that maternal diabetes hyperglycemia preceding embryo implantation exerted its action on neural crest precursor cells at the beginning of the organogenic period disturbing proliferation and/or cell migration. We also speculate that the maternal hyperglycemia had a differential effect on offspring which depended on the intrinsic susceptibility of the embryo to a hyperglycemic intrauterine environment. In a small group of highly susceptible embryos (13%) maternal diabetes caused severe craniofacial malformations which led to intrauterine death. In contrast, in the remaining population (87%), with low susceptibility to hyperglycemia, maternal diabetes altered the intrauterine development timeline, restricted embryo-fetal growth, and delayed the remodeling and maturation primarily of the structures derived from neural crest cells. Evidences exist of a close relationship of dependence of the normal development of the neural tube and the skull [39]. This fact indicates that the delay of the development of the craniofacial structures in F21 of DRs may be accompanied by a delay in the development and maturation of the neural tube. Although craniofacial deficiencies were counterbalanced in NB pups during the two days in which the gestation was continued in the DRs, we ignore the consequences in the short and medium term in learning, behavior, and quality of the life of the offspring. The effect of maternal diabetes in the limb development of the low susceptible embryos may be related to delay of morphogenetic mechanisms involving programmed cell death. The most obvious consequences were the asynchrony in occurrence of interdigital membrane death and delay in endochondral ossification of digits.

## 5. Conclusions

The present study demonstrates the effect of severe diabetic status from the beginning of pregnancy that reproduced maternal and fetal outcomes of pregnant women presenting uncontrolled clinical diabetes. Delay of the morphologic and functional maturation of structures mainly derived from neural crest cells may be accompanied by an asynchrony in the development and maturation of the neural tube. Consequently our findings provide valuable information to analyze with a new vision the cognitive and behavioral processes in children of diabetic women with the purpose for designing appropriate strategies to enable children exceptional achievements in learning.

## Figures and Tables

**Table 1:** Litter characteristics.

	CR	DR
Gestation period	21 days	23 days
Number of pups	10.4 (2.3)	5.6 (2.70)^*^
Number of resorptions	0.6 (0.54)	3.2 (1.30)^*^
Intrauterine deaths (%)	0	4^*^

Data are expressed as means (standard deviations) and percentages. ^*^ *P* < 0.001 in control rats (CR) compared with diabetic rats (DR).

**Table 2:** Glycemia during pregnancy in mg/dL.

	Glycemia in pregnant rats to obtain F21		Glycemia in pregnant rats to obtain NB	
	CR (*n* = 10)	DR (*n* = 15)	CR (*n* = 10)	DR (*n* = 15)
Day 0	89.60 (6.88)	88.33 (11.02)	84.50 (15.25)	93.00 (5.00)
Day 7	99.14 (11.85)	371.00 (48.04)^*^	98.14 (8.75)	352.22 (51.76)^*^
Day 12	92.86 (6.17)	391.86 (62.34)	93.00 (5.51)	429.44 (55.37)
Day 21	105.29 (8.77)	412.71 (68.54)	90.14 (4.78)	438.52 (46.14)
Day 23				447.56 (57.06)

Data are expressed as means (standard deviations). ^*^ *P* < 0.001 in streptozotocin-induced diabetic rats compared with controls from Day 7 of pregnancy.

**Table 3:** Body weight of pregnant rats (in grams).

	Weight of pregnant rats to obtain F21		Weight of pregnant rats to obtain NBs	
	CR (*n* = 10)	DR (*n* = 15)	CR (*n* = 10)	DR (*n* = 15)
Day 0	276.28 (27.53)	268.1 (12.06)	284.85 (24.75)	253.33 (8.70)
Day 5	285.14 (29.14)	275.4 (11.77)	300.00 (32.02)	263.44 (11.04)
Day 7	299.57 (25.65)	286.7 (16.64)	318.00 (26.00)	268.77 (17.37)
Day 12	325.14 (28.43)	305.6 (22.18)	345.57 (19.20)	284.11 (19.89)
Day 15	361.14 (33.74)	323.9 (45.39)^*^	383.00 (23.04)	305.55 (27.40)^*^
Day 21	395.0 (36.48)	348.6 (42.11)	418.85 (25.95)	337.11 (21.54)
Day 23				350.34 (16.04)

Data are expressed as means (standard deviations) of control rats (CR) and diabetic rats (DR). ^*^ *P* < 0.05 in streptozotocin-induced diabetic rats compared with controls.

**Table 4:** Maternal hyperglycemia effect on pup somatometry of control rats (CR) and diabetic rats (DR).

	F21			NB		
	CR	DR	*P* value	CR	DR	*P* value
Weight	6.27 (0.62)	3.61 (0.81)	0.000	6.92 (0.50)	6.72 (0.64)	0.032
CRL	4.05 (0.19)	3.30 (0.39)	0.000	4.40 (0.16)	4.36 (0.24)	0.30
NO	1.67 (0.08)	1.34 (0.21)	0.000	1.70 (0.10)	1.73 (0.09)	0.151
Tail	1.53 (0.12)	1.34 (0.13)	0.000	1.64 (0.11)	1.66 (0.14)	0.201
Waist ⌀	1.45 (0.21)	1.13 (0.10)	0.000	1.42 (0.14)	1.41 (0.12)	0.680

Data are expressed as means (standard deviations) of the various morphometric parameters using *P* < 0.05 for comparisons. CRL = crown-rump length, NO = nasooccipital length, tail = tail length, and waist ⌀ = waist diameter.
